# Impact of breast tumor size discrepancy between contrast-enhanced and conventional ultrasonography on axillary node metastasis: a retrospective cohort study

**DOI:** 10.1186/s12885-025-15167-9

**Published:** 2025-11-05

**Authors:** Tomohiro Oshino, Hirokazu Shimizu, Megumi Sato, Mutsumi Nishida, Tatsunori Horie, Satonori Tsuneta, Fumi Kato, Mitsuchika Hosoda, Isao Yokota, Kohsuke Kudo, Masato Takahashi

**Affiliations:** 1 Department of Breast Surgery, Hokkaido University Hospital, Kita 14 Nishi 5, Kita-ku, Sapporo, Hokkaido Japan; 2 Department of Orthopedic Surgery, Faculty of Medicine, Graduate School of Medicine, Hokkaido University, Sapporo, Hokkaido Japan; 3 Diagnostic Center for Sonography, Hokkaido University Hospital, Sapporo, Hokkaido Japan; 4 Department of Diagnostic and Interventional Radiology, Hokkaido University Hospital, Sapporo, Hokkaido Japan; 5 Department of Radiology, Graduate School of Dental Medicine, Hokkaido University, Sapporo, Hokkaido, Japan; 6 Department of Radiology, Jichi Medical University Saitama Medical Center, Saitama, Saitama Japan; 7 Department of Biostatistics, Hokkaido University Graduate School of Medicine, Sapporo, Hokkaido Japan; 8 Department of Diagnostic Imaging, Graduate School of Medicine, Hokkaido University, Sapporo, Hokkaido Japan

**Keywords:** Contrast-enhanced ultrasonography, Size discrepancy, Breast cancer, Lymph node metastasis

## Abstract

**Background:**

Conventional ultrasonography (cUS) and contrast-enhanced ultrasonography (CEUS) are used to evaluate breast cancer tumors and axillary lymph nodes (ALN), by which the treatment strategy for breast cancer is determined. A breast tumor size discrepancy on CEUS compared with cUS is often observed, for which the reasons are unclear. We hypothesized that this discrepancy reflects the metastatic potential, and this study investigated the association between size discrepancies on cUS and CEUS in relation to ALN metastasis in breast cancer.

**Methods:**

This retrospective study enrolled 259 patients who underwent surgery for breast cancer after preoperative cUS and CEUS examinations. Patients were grouped into a DISCR (i.e., tumor size discrepancy ≥ 4.0 mm between CEUS and cUS measurements) and non-DISCR group. The primary outcome was ALN metastasis, defined by pathological evaluation. Secondary outcomes were the 5-year recurrence-free survival rates.

**Results:**

There were 94 patients in the DISCR and 165 in the non-DISCR groups. No tumor size differences measured by cUS were observed between two groups (*p* = 0.82), whereas the DISCR group had a significantly higher rate of ALN metastasis (*p* < 0.01). Multivariate analyses showed a discrepancy of ≥ 4.0 mm was a risk for ALN metastasis (odds ratio: 5.838, 95% confidence interval [CI]: 2.408–14.155). The 5-year recurrence-free survival rate was lower in the DISCR (0.750, 95% CI: 0.632–0.868) than in the non-DISCR (0.924, 95% CI; 0.870–0.978) group.

**Conclusion:**

An increase in contrast-enhanced ultrasonography tumor size is helpful for assessing axillary lymph node metastasis and prognosis.

**Supplementary Information:**

The online version contains supplementary material available at 10.1186/s12885-025-15167-9.

## Background

Breast cancer is the most common malignancy in women, resulting in the second highest mortality rate [1]. It is crucial to evaluate axillary lymph node (ALN) metastasis in breast cancer, as ALN metastasis in early breast cancer affects prognosis [2]. However, axillary dissection and sentinel lymph node biopsy are invasive, and unacceptable complications may occur [3]. Therefore, ALN surgery for breast cancer has recently been decreasing, and case selection for surgical ALN reduction often requires imaging evaluation of suspected metastases [4, 5]. An accurate and non-invasive method for predicting ALN status without axillary surgery is warranted.

Conventional ultrasonography (cUS) is the method of choice for assessing breast cancer and ALN due to a lower cost and ease of use [6]. A recent report introduced a radiomics model to predict the ALN metastasis using cUS images of primary breast cancer which showed diagnostic performance [7]. Contrast-enhanced ultrasonography (CEUS) uses contrast agents to enhance the contrast between vessels and surrounding tissues. It can provide information on microcirculatory perfusion in the lesion and features such as the number, thickness, shape, and distribution of blood vessels, which cannot be detected by cUS ([8, 9]). Adding CEUS examinations to primary breast cancer or ALN can improve diagnostic performance in determining ALN status ([10, 11]).

In primary breast cancers, a size increase with CEUS is more likely in malignant cancers than in benign lesions [12]. Previous studies have shown that vascular remodeling at the invasive front of malignant lesions plays a crucial role in metastasis ([13, 14]). We have hypothesized that the size discrepancy between CEUS and cUS might reflect the metastatic potential of the lesion. The aim of this study was to investigate the association of tumor size increase observed when adding CEUS to cUS and ALN metastasis in early breast cancer.

## Materials and methods

### Study design

**Fig. 1:**
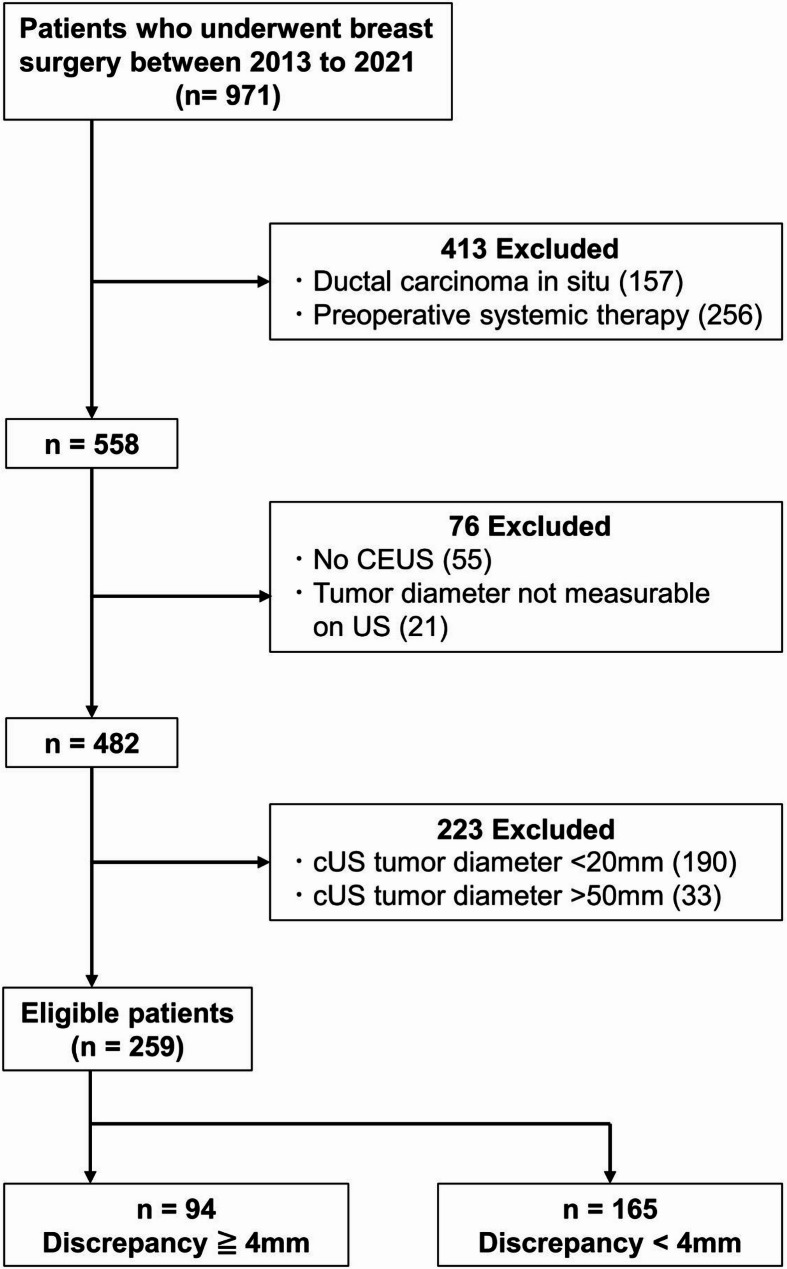
Study design cUS: conventional US, CEUS: Contrast enhanced ultrasonography

This retrospective, single-center study was approved by the ethics committee of our hospital (020–0392), and the written informed consent was waived. All studies were performed in accordance with the relevant guidelines and regulations. We reviewed the records of 971 patients who underwent surgery for early breast cancer (clinical stage 0–3) at our hospital between 2013 and 2021 (Fig. 1) and excluded those with ductal carcinoma in situ, preoperative systemic therapy, absence of preoperative CEUS, and unqualified US images. After exclusion, cUS images were reviewed and patients with breast cancer tumor size by cUS ranged from 20 mm to 50 mm were enrolled in this study. Most of the cases reported in this study are part of those in previous study [11]. They were grouped into DISCR (discrepancy ≥ 4.0 mm in tumor size by adding CEUS) and non-DISCR. The threshold of 4.0 mm was set according to a previous report [12]. The medical records of the enrolled patient were reviewed.

### US protocols

Preoperative cUS and CEUS of breast cancer cases were performed by two sonographers specializing in breast cUS and CEUS for 5 years or more. CEUS was performed by administering Sonazoid^®^ (GE HealthCare Pharma, Tokyo, Japan) 0.015 mL/kg/body by intravenous injection, and the breast tumor was evaluated in the cross section with the maximal contrast accumulation. Images with the highest contrast accumulation in the CEUS series were selected for this study. Contrast accumulation reached its peak in arterial phases within 60 s after contrast injection.

### Comparison of lesion maximum diameter

**Fig. 2:**
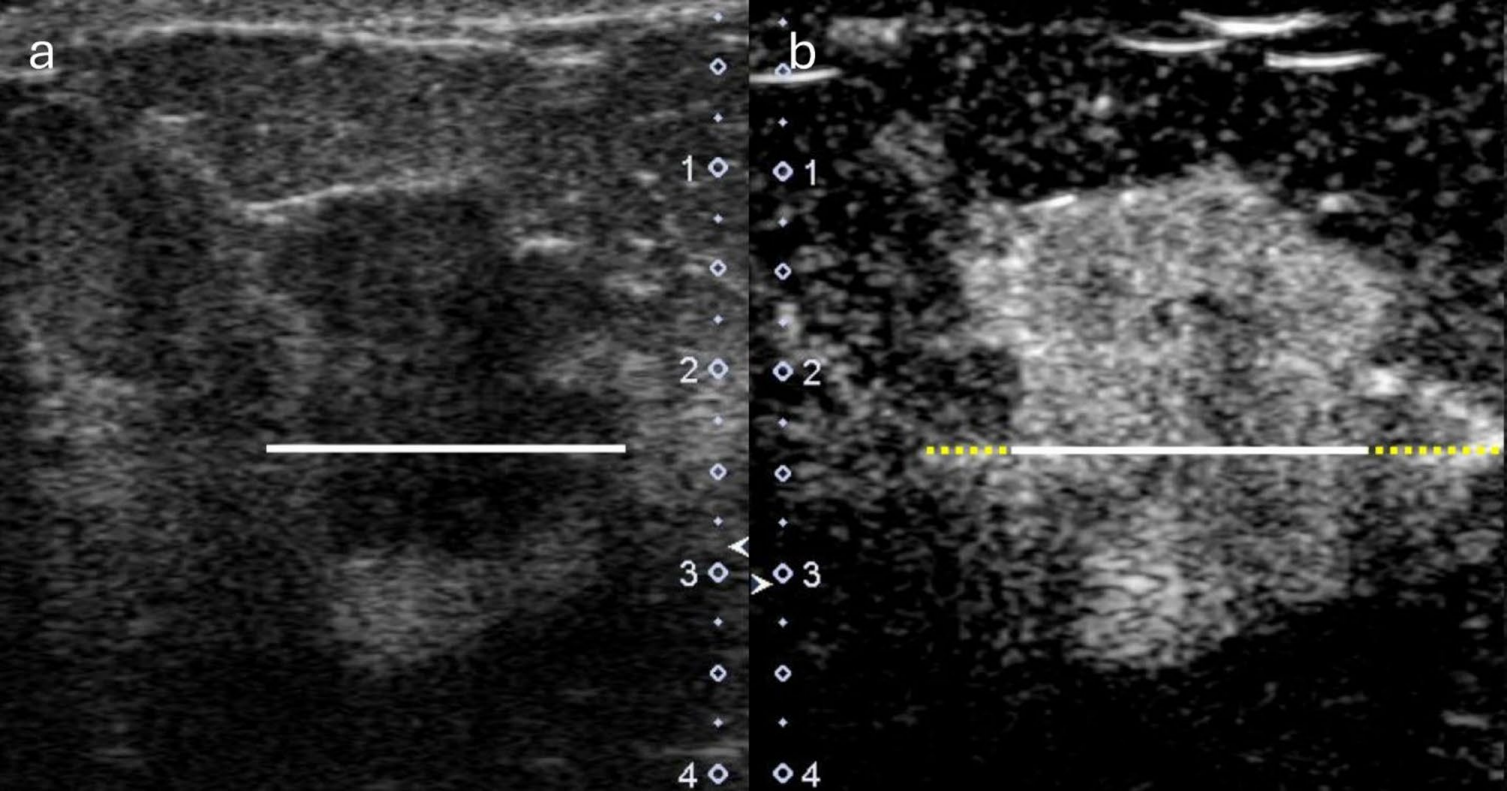
Representative images of patients grouped into DISCR. The patients with axillary node metastasis- positive breast cancer, who were a 65-year-old female (**a**) Diameter measurements by conventional ultrasonography, (**b**) diameter measurements by contrast-enhanced ultrasonography. The white solid line indicates the tumor size evaluated by conventional ultrasonography (21 mm). The yellow dashed line indicates the size discrepancy (6 mm). Because the discrepancy is ≥ 4 mm, this patient is categorized as DISCR

All imaging features were evaluated by two breast surgeons with 9 and 34 years of experience, respectively. Interobserver agreement was tested by evaluating 80 randomly selected patients. A comparison of the maximum lesion diameter between cUS and CEUS was performed according to previous reports [12, 15]. As cUS shows different patterns of tumor appearance, the measurements were performed in the following ways: (i) for lesions with circumscribed margins, the hypoechoic core of the tumors were measured; (ii) for lesions with microlobulations, angulations, or spiculations, the measurements included microlobulations, angulations, spiculations, or the distance from the tip of one spicule to the tip of an opposing spicule. The maximum lesion diameter on CEUS was measured manually from the maximum-enhancement images. Enhancement was defined as an increase in lesion echoes after contrast agent injection compared to cUS. All enhancement components within and adjacent to the tumor region on cUS were included. Representative images are shown in Fig. 2.

### Imaging features in CEUS

For primary breast tumors, cUS assessment included the BI-RADS category [16] and the long and short diameters. The CEUS evaluation of primary tumors involved determining the long and short diameters and the presence of nine specific findings [17]: A1) enhanced time; A2) enhanced intensity A3) enhanced direction; A4) internal homogeneity; A5) margin of the lesion; A6) shape of the lesion; A7) ring-like enhancement; A8) scope of the lesion; and A9) perfusion defect. For detailed findings see the supplementary table.

### Univariate and multivariate analysis

Univariate analyses were performed to compare the characteristics and ALN metastasis of the DISCR and non-DISCR groups.

Multivariate analysis was performed to predict ALN metastasis according to discrepancies and CEUS findings. The equation for multiple logistic regression is

$$\begin{aligned} \mathrm y\;=&\;{\mathrm\beta}_0\;+\;{\mathrm\beta}_1{\mathrm x}_1\;\\&+\;{\mathrm\beta}_2{\mathrm x}_2\;+\;{\mathrm\beta}_3{\mathrm x}_3\;+...\\&+\;{\mathrm\beta}_{10}{\mathrm x}_{10}\;+\;\mathrm\varepsilon\end{aligned}$$

where y is the predictive value for predicting ALN metastasis (0–1), β_0_ is intercept value, x_i_ are the ten independent variables (discrepancy and A1-A9), β_i_ are the estimated regression coefficients of respective independent variables, ε is the model error. A1–A9 are defined as binary variables (e.g., If the positive findings shown in supplementary table are present, the value is 1; otherwise, it is 0.).

The discriminatory ability of the model was assessed using a receiver operating characteristic curve, and the area under the curve (AUC) was calculated to evaluate its performance.

### Outcomes

The primary outcome was the pathological evaluation of ALN metastasis. Secondary outcomes, including treatment strategies for each group, 60-month recurrence-free survival (defined as the time from surgery to first locoregional recurrence, including regional lymph nodes or distant recurrence), and overall survival (defined as the time from surgery to death from any cause), were evaluated.

### Statistical analysis

Categorical variables were evaluated using the chi-square test for two groups, whereas continuous variables were analyzed using an independent t-test. *P*-values were adjusted for multiple comparisons using the Bonferroni correction to control the family-wise error rate. Logistic regression analysis was used for multivariate analysis. Survival rates were visualized using the Kaplan–Meier method. All statistical analyses were performed using Bell Curve for Excel version 4.02 or EZR version 1.68, with a significance level set at 0.05.

## Results

### Patients and clinicopathological characteristics

From 971 patients who underwent surgery for early breast cancer (clinical stage 0–3) we excluded 489 patients. After these exclusions, 482 cUS images were reviewed, and 190 patients with tumor diameters < 20 mm (pN+, *n* = 30) and 33 patients with tumor diameters > 50 mm (pN+, *n* = 16) were further excluded. In total, 259 patients were enrolled in this study (Fig. 1). The baseline characteristics of the enrolled patients (median age 64, range 30–90) are shown in Table 1. The median pathological invasive diameter was 18 (range 0.0–100.0.0.0). The estrogen receptor positivity rate was 83.8% (217/259), the progesterone receptor positivity rate was 74.9% (194/259), and the HER2 positivity rate was 12.8% (33/258, 1 was not evaluated). The median Ki-67 index was 22.6% range (0.0–98.5.0.5, 9 cases were not evaluated), and the rate of invasive ductal carcinoma was 82.6% (214/259). The kappa values for evaluating CEUS dataset findings were 0.96 (0.94–0.97) for inter-observer agreement (*p* < 0.001, kappa test). No differences were observed in tumor size between the DISCR and non-DISCR groups on cUS. The CEUS diameter was significantly higher in the DISCR group (*p* < 0.01) (Table 2).

**Table 1:** Patient baseline characteristics

Characteristics	*n* = 259
Age (years)^a^	64 [30–90]
Pathological invasive diameter (mm)^a^	18 [0.0–100.0.0.0]
Estrogen receptor	
Positive	217 (83.8%)
Negative	42 (16.2%)
Progesterone receptor	
Positive	194 (74.9%)
Negative	65 (25.1%)
HER2	
Positive	33 (12.8%)
Negative or equivocal	225 (87.2%)
Ki-67 (%)^a^	22.6 [0.0–98.5.0.5]
Tumor type	
Invasive ductal carcinoma	214 (82.6%)
Invasive lobular carcinoma	23 (8.9%)
Other types	22 (8.5%)

*HER2* human epidermal growth factor receptor 2

a: median [range]

**Table 2:** Association of size discrepancy with lesion diameter and imaging features assessed using cUS and CEUS

	DISCR (*n* = 94)	non-DISCR (*n* = 165)	Raw *p*-value	Bonf adjusted *p*-value
Conventional US diameter^a^ (mm)	29 [20–50]	29 [20–49]	0.82	0.99
Conventional US BI-RADS			0.089	0.99
0–3	7 (7.4%)	8 (4.8%)		
4	42 (44.7%)	109 (65.7%)		
5	45 (47.9%)	49 (29.5%)		
CEUS diameter^a^ (mm)	39 [25–65]	28 [18–52]	< 0.001*	< 0.001*
CEUS findings of primary breast tumor				
A1: Enhanced time; earlier	85 (90.4%)	146 (85.0%)	0.54	0.99
A2: Enhanced intensity; enhanced	94 (100%)	158 (95.2%)	0.030*	0.36
A3: Enhanced direction; centripetal	84 (89.4%)	145 (87.3%)	0.63	0.99
A4: Internal homogeneity of the lesion; heterogeneous	69 (73.4%)	130 (78.3%)	0.37	0.99
A5: Margin of the lesion; not clear	31 (33.0%)	75 (45.2%)	0.062	0.74
A6: Shape of the lesion; irregular	31 (33.0%)	77 (46.4%)	0.040*	0.48
A7: Ring-like enhancement; present	13 (13.8%)	6 (3.6%)	0.0025*	0.03*
A8: Scope of the lesion; extended	79 (84.0%)	121 (72.9%)	0.046*	0.55
A9: Perfusion defect; present	52 (55.3%)	75 (45.2%)	0.12	0.99

DISCR, group with a discrepancy ≥ 4 mm; Bonf, Bonferroni, *cUS*, conventional ultrasonography, *CEUS *contrast-enhanced ultrasonography, *BI-RADS *Breast imaging reporting and data system

*Statistically significant,

a: median [range]

### Association of DISCR and US features of breast tumors with ALN metastasis

**Fig. 3:**
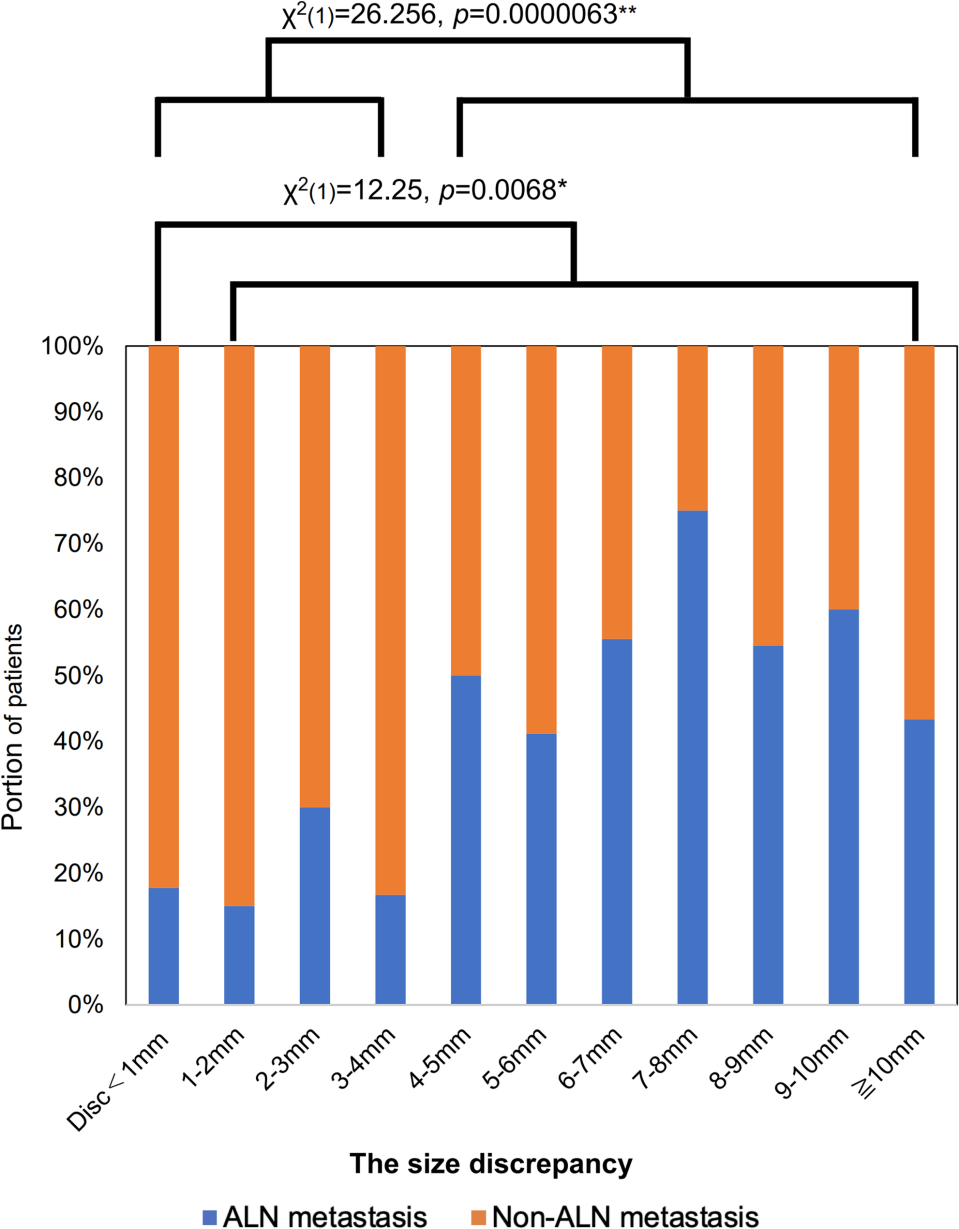
Association of the size discrepancy with axillary lymph node metastasis. The histogram shows the proportion of patients with discrepancies for each size. Statistical significance between discrepancies of < 4 mm was evaluated using the chi-square test ALN: axillary lymph node

**Fig. 4:**
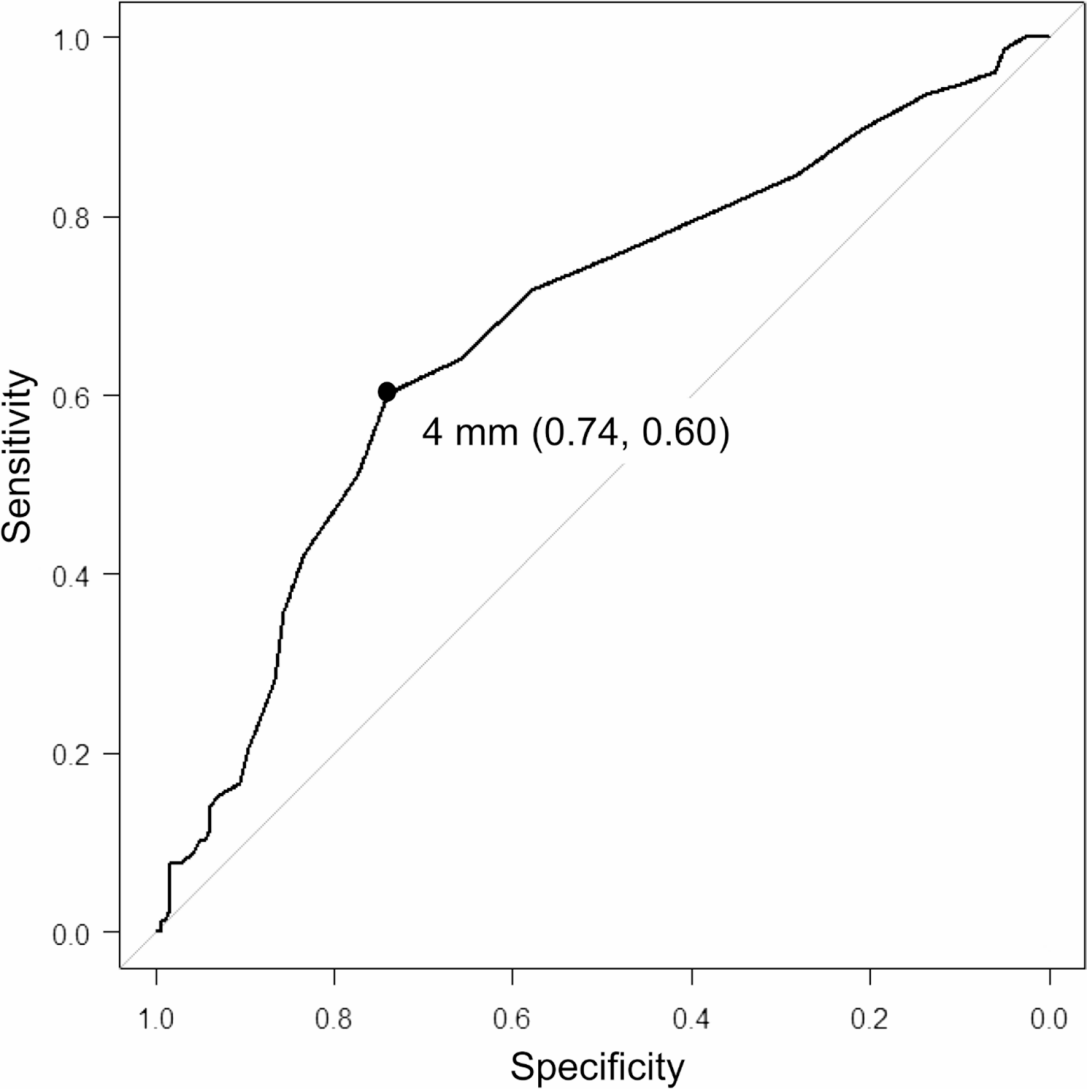
Receiver Operating Characteristic Validation of the 4-mm discrepancy Cutoff. Sensitivity, 0.74; specificity, 0.60; area under the receiver operating curve, 0.677 [95% CI, 0.604–0.749]

**Fig. 5:**
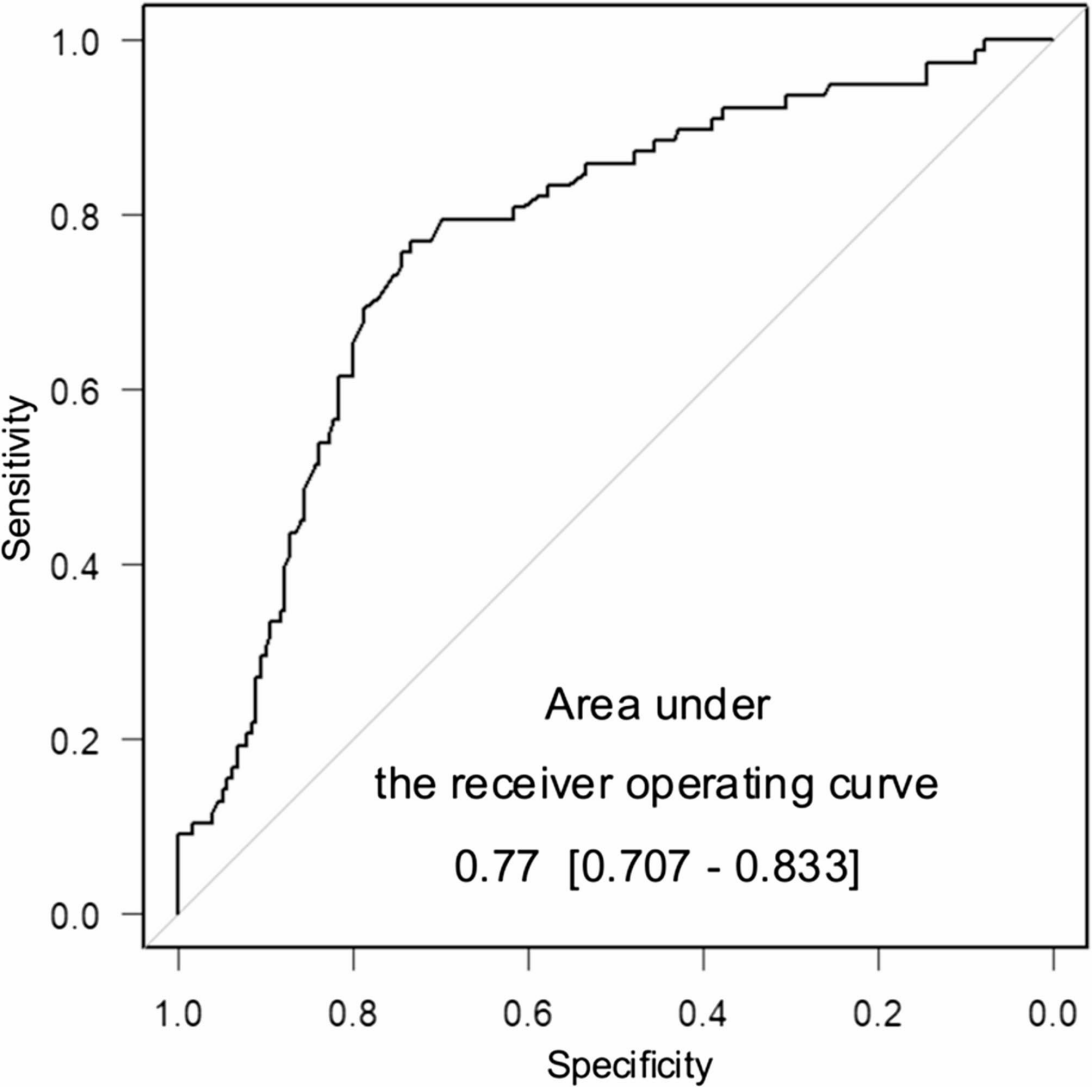
Contrast-enhanced ultrasonography-based model performance to predict axillary lymph metastasis

ALN metastasis was evaluated for each 1-mm increment in discrepancy and differences in ALN metastasis were assessed according to the presence or absence of discrepancy and whether the discrepancy was ≥ 4 mm or not. The DISCR group had ALN metastasis in 50% of patients (47/94), whereas the non-DISCR group had ALN metastasis in 19% of patients (31/165). Fig. 3 shows the proportion of ALN metastasis according to the size of the discrepancy (in mm). Patients with DISCR had a significantly higher rate of ALN metastasis than those without DISCR (*p* < 0.001). Receiver operating characteristic curve analysis supported a cutoff value of 4 mm (sensitivity, 0.74; specificity, 0.60; AUC, 0.677 [95% CI, 0.604–0.749]) (Fig. 4). Among the qualitative evaluations of CEUS listed in Table 2, several factors were associated with DISCR: A2, enhanced intensity (*p* = 0.030); A6, shape of the lesion (*p* = 0.040); A7, ring-like enhancement (*p* < 0.01); and A8, scope of the lesion (*p* = 0.046). After Bonferroni adjustment for multiple comparisons, only A7 remained significant (*p* = 0.03). The prediction model based on CEUS findings, in which the discrepancy combined with qualitative factors (A1-A9) for ALN metastasis, showed moderate performance, with an area under the curve of 0.77 [95% confidence interval [CI]; (0.707–0.833) (Fig. 5).

### Multivariate analyses

Sixteen cases with missing explanatory variable values were excluded from the multivariate analysis. These included nine with missing Ki-67 data, eight with missing tumor grade data, one with missing HER2 data, and one with missing A8 data; three cases had overlapping missing values. Among the excluded cases, three were in the DISCR group with ALN metastasis, three were in the non-DISCR group with ALN metastasis, and 10 were in the non-DISCR group without ALN metastasis. The potential effect of these exclusions on our findings was considered minimal. The multivariate analysis showed that DISCR was an independent risk factor for ALN metastasis (odds ratio [OR]: 5.838, 95% CI, 2.408–14.155), whereas CEUS diameter was not a significant factor (OR, 0.984; 95% CI, 0.939–1.030) (Table 3). Invasive diameter by pathological diagnosis was another independent risk factor (OR:1.073, 95% CI, 1.034–1.112). The multivariate analysis which regarded discrepancy as a continuous variable also showed that the discrepancy was an independent risk factor (OR:1.106, 95% CI, 1.022–1.197) (Table 4). Based on the multivariate logistic regression analysis, the multivariate prediction model was formulated as follows:

**Table 3:** Multivariate analysis for axillary lymph node metastasis with discrepancy (qualitative ≥ 4.0 mm)

		ALN metastasis		
	Odds ratio	Lower95% CI		Upper95% CI
Discrepancy (mm)*	1.106	1.022		1.197
CEUS diameter	0.986	0.937		1.039
Invasive diameter by pathological diagnosis*	1.068	1.031		1.106
Tumor grade	0.779	0.390		1.557
Estrogen receptor	1.003	0.989		1.018
Progesterone receptor	0.994	0.982		1.007
HER2	0.900	0.668		1.213
Ki67	0.998	0.974		1.023
Venous invasion	1.268	0.527		3.052
Lymphatic invasion*	5.033	2.077		12.196
A2: Enhanced intensity; enhanced	0.896	0.048		16.843
A6: Shape of the lesion; irregular	1.037	0.437		2.458
A7: Ring-like enhancement; positive	3.735	0.851		16.391
A8: Scope of the lesion; extended	1.584	0.508		4.939

**Table 4:** Multivariate analysis for axillary lymph node metastasis with discrepancy (quantitative)

		ALN metastasis	
	Odds ratio	Lower 95% CI	Upper 95% CI
Discrepancy (4.0 mm or more)*	5.838	2.408	14.155
CEUS diameter	0.984	0.939	1.030
Invasive diameter by pathological diagnosis*	1.073	1.034	1.112
Tumor grade	0.772	0.375	1.589
Estrogen receptor	1.003	0.988	1.018
Progesterone receptor	0.994	0.981	1.007
HER2	0.891	0.648	1.227
Ki67	0.997	0.971	1.023
Venous invasion	1.276	0.511	3.186
Lymphatic invasion*	5.011	1.970	12.748
A2: Enhanced intensity; enhanced	0.728	0.038	14.022
A6: Shape of the lesion; irregular	0.878	0.359	2.144
A7: Ring-like enhancement; positive	4.021	0.909	17.785
A8: Scope of the lesion; extended	1.445	0.453	4.611

Adjusted for age, tumor type, breast imaging reporting, and data system, A1, A3, A4, A5, and A9

*ALN *axillary lymph node, *CEUS *contrast-enhanced ultrasonography, *HER2 *human epidermal growth factor receptor 2

*Statistically significant

Prediction Score = − 4.573 + 0.0862 × [Discrepancy (mm)] + 1.515 × [A1] + 0.0133 × [A2] + 0.794 × [A3] + 0.250 × [A4] – 0.465 × [A5] + 0.244 × [A6] + 1.309 × [A7] + 1.097 × [A8] + 0.362 × [A9].

### Treatments and prognosis

The therapeutic strategies are listed in Table 5. The total mastectomy rate was 84.0% (76/94) in the DISCR group and 81.2% (134/165) in the non-DISCR group (*p* = 0.646). The axillary dissection rate was 27.7% (26/94) in the DISCR group and 13.9% (23/165) in the non-DISCR group (*p* = 0.012). The chemotherapy rates were 44.7% (42/94) for the DISCR group and 27.3% (45/165) for the non-DISCR group (*p* < 0.01). The postmastectomy radiotherapy rates were 11.7% (11/94) for the DISCR group and 3.6% (6/165) for the non-DISCR group (*p* = 0.012).

**Table 5:** Therapeutic strategies and treatment modalities in DISCR and non-DISCR groups

Treatment	DISCR (*n* = 94)	non-DISCR (*n* = 165)	*p*-value
Breast operation			0.65
Bt	79 (84.0%)	134 (81.2%)	
Bp	15 (16.0%)	31 (18.8%)	
Axillary operation			0.012*
SN	68 (72.3%)	142 (86.1%)	
Ax	26 (27.7%)	23 (13.9%)	
Endocrine therapy			0.64
Yes	68 (72.3%)	125 (75.8%)	
No	25 (26.6%)	40 (24.2%)	
Unknown^a^	1 (1.1%)	0	
Chemotherapy			0.0036*
Yes	42 (44.7%)	45 (27.3%)	
No	51 (54.3%)	120 (72.7%)	
Unknown^a^	1 (1.1%)	0	
Anti HER2 therapy			0.44
Yes	10 (10.6%)	13 (7.9%)	
No	83 (88.3%)	152 (92.1%)	
Unknown^a^	1 (1.1%)	0	
PMRT			0.012*
Yes	11 (11.7%)	6 (3.6%)	
No	83 (88.3%)	159 (96.4%)	

*DISCR *group with a discrepancy ≥ 4 mm, *Bt *total mastectomy, *Bp* partial mastectomy, *Sn *sentinel lymph node biopsy, *Ax *axillary dissection, *HER2 *human epidermal growth factor receptor 2, *PMRT* postmastectomy radiotherapy

a: The same patient who was transferred to another hospital immediately after surgery

**Fig. 6:**
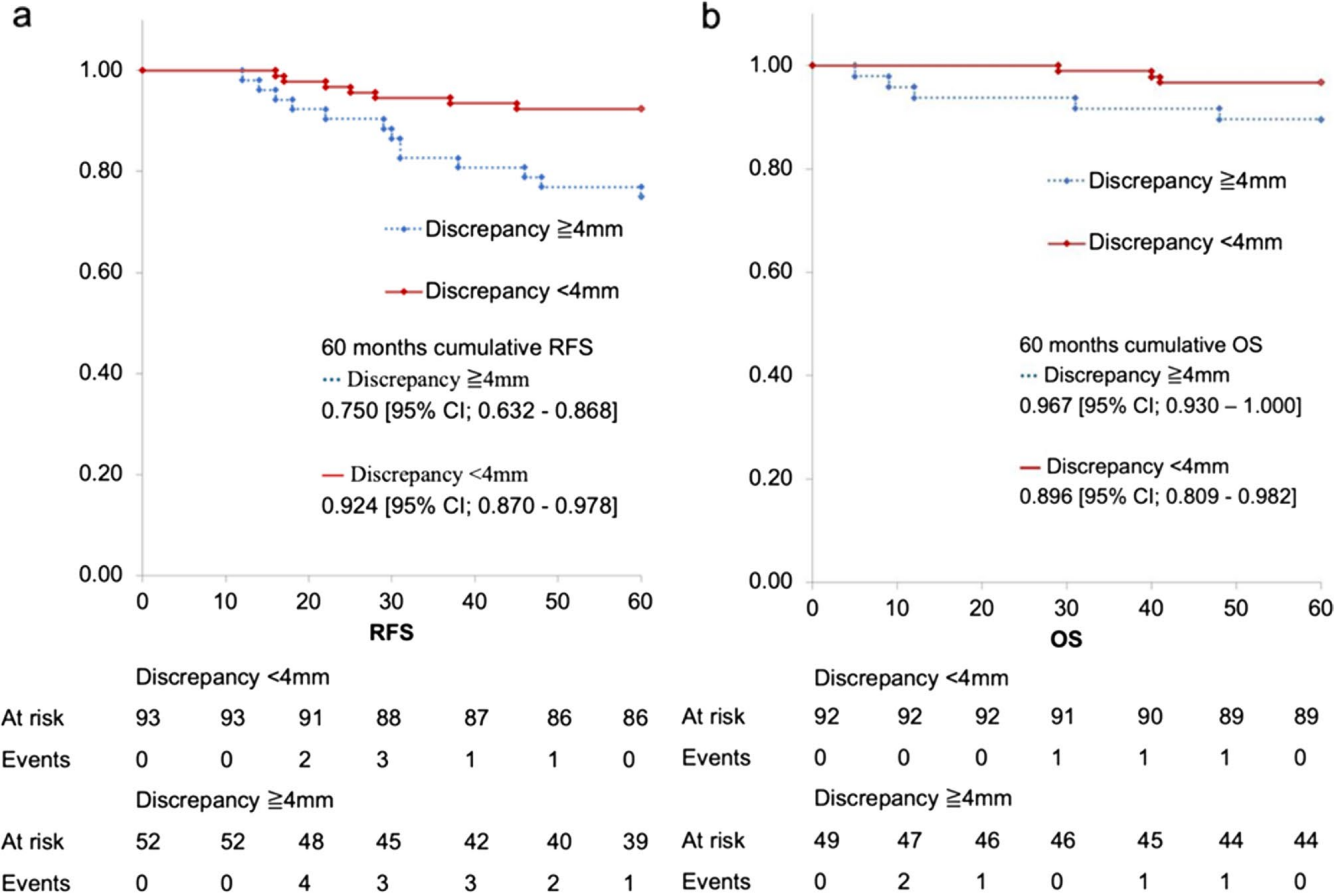
Prognosis. Survival analysis represents the proportion of (**a**) recurrence-free survival (RFS) and (**b**) overall survival (OS)

The median follow-up period was 73 months (range; 5–125 months). Locoregional recurrence (including regional lymph nodes) was observed in 10 patients (DISCR, *n* = 5; non-DISCR, *n* = 5). Distant recurrence occurred in 19 patients (DISCR group, *n* = 12; non-DISCR group, *n* = 7). Death from any cause was recorded in 11 patients, including six breast cancer-specific deaths (DISCR, *n* = 2; non-DISCR, *n* = 4), four non-breast cancer deaths (DISCR, *n* = 2; non-DISCR, *n* = 2), and one non-breast cancer death following distant recurrence (DISCR, *n* = 1). The 5-year recurrence-free survival rate was significantly lower in the DISCR (0.750, 95% CI: 0.632–0.868) than non-DISCR (0.924, 95% CI; 0.870–0.978) group (Fig. 6a). The 5-year overall survival rate was 0.869 (95% CI: 0.809–0.982) in the DISCR group and 0.967 (95% CI; 0.930–1.000.930.000) in the non-DISCR, without a significant difference (Fig. 6b).

## Discussion

This study demonstrated that the size discrepancy in breast cancer between contrast-enhanced ultrasonography and conventional ultrasonography was associated with axillary lymph node metastasis and prognosis. Multivariate analyses showed that size discrepancy was an independent risk factor for axillary lymph node metastasis, whereas diameter on contrast-enhanced ultrasonography was not a risk factor. This indicates that the size discrepancy between contrast-enhanced and conventional ultrasonography reflects metastatic potential. Notably, a difference was observed in RFS, but no difference was detected in OS. In the current era, long-term survival after recurrence has become common in breast cancer; consequently, many patients remain alive after recurrence, which may obscure detectable differences in OS.

If the discrepancy between CEUS and cUS tumor diameters is predictive of ALN metastasis, it may be useful in clinical practice. CEUS has mainly been used to differentiate benign from malignant lesions [18] because it has diagnostic capabilities comparable to those of contrast-enhanced breast MRI [19]. Although previous studies have reported an association between CEUS tumor size itself or tumor area and ALN metastasis [10], no malignancy-specific imaging features have been identified, limiting its clinical applicability [20]. This study demonstrated the utility of CEUS in predicting ALN metastasis not by tumor size alone but by its discrepancy compared to cUS. Such discrepancy may contribute to predicting ALN metastasis. Moreover, measurement of tumor discrepancy is easier than for tumor area by CEUS, making it more suitable for clinical use. Compared with a previous report [12] that examined the differentiation between benign and malignant breast lesions, our study evaluated a larger cohort of breast cancer patients using a similar methodology and investigated the association of size discrepancy with the presence of ALN metastasis. Preoperative imaging assessment of ALN metastasis is critical for treatment planning, and its ability to predict ALN status has substantial clinical significance. In the previous report [12], the discrepancy threshold was tumor size-dependent: 4 mm for tumors ≥ 17 mm and 3 mm for tumors < 17 mm. Although tumors < 20 mm were excluded from the present study, future studies should include a broader tumor size range and adopt size-specific thresholds.

In clinical settings, preoperative prediction of ALN status is crucial because omission of surgical axillary staging has been actively investigated [21]; however, missed lymph node metastasis may affect not only local treatment but also drug therapy and prognosis [22–25]. To this end, CEUS can help surgeons avoid inappropriate omission of sentinel lymph node biopsies. On the other hand, CEUS is a time-consuming examination, and it is not practical to perform it for all patients undergoing breast cancer surgery. Although CEUS provides additional diagnostic information, its routine use in clinical practice may be limited by cost, availability, and operator expertise. In addition to financial considerations, CEUS often requires additional examination time and dedicated staff for contrast administration and image interpretation, which may further affect its feasibility and integration into busy clinical workflows. In clinical workflows, CEUS could be reserved for patients with breast cancer who show no evidence of ALN metastasis on cUS, CT, or MRI (cN0), and for whom omission of sentinel lymph node biopsy is being considered. Further prospective studies should be performed investigating the omission of the superficial axillary staging based on CEUS-based assessments.

Although it is a known clinical phenomenon that increases in size are observed with CEUS, the underlying mechanisms have not been clarified. Although our hypothesis regarding the association between size discrepancy and metastatic potential was consistent with the results of the present study, the mechanisms are not yet clarified. In tumor biology, tumor cells migrate into and invade the surrounding tissue by forming an invasive front [26]. A xenograft model with 3-dimensional microvascular data showed enrichment of angiogenesis in the invasive front compared with the tumor core [14]. The increase in tumor size when CEUS is added might reflect that parts of the invasive front have genetic alterations [27], adaptation to the host environment [28], metabolic differences [29], epithelial-mesenchymal transition, or stem cell-like features [30]. These are hypotheses only, and further studies are needed to prospectively elucidate the characteristics of edge areas where blood vessels are enriched.

Our study has several limitations. First, this was a single-center, retrospective study. Furthermore, tumors > 50 mm were excluded due to measurement difficulties, and tumors < 20 mm were excluded because of the low frequency of ALN metastasis. There may have been recall or selection bias. Second, the fact that the image qualifying and imaging features were determined by the physician’s judgment may have introduced subjective bias. Operator and equipment dependence in CEUS measurements could also contribute to observer bias. Third, we did not analyze the size discrepancy using other modalities, such as MRI. This may have affected our results.

## Conclusions

The discrepancy in breast cancer size between conventional ultrasonography and contrast-enhanced ultrasonography was an independent risk factor for axillary lymph node metastasis. It is also associated with prognosis. From a biological perspective, this discrepancy might play a crucial role in the mechanism of metastasis.

## Supplementary Information

Supplementary Material 1.

## Data Availability

The data supporting the findings of this study are available from the corresponding author upon request.

## Abbreviations

ALN: Axillary lymph node
cUS: Conventional ultrasonography
CEUS: Contrast-enhanced ultrasonography
DISCR: Group with a discrepancy ≥ 4 mm
AUC: Area under the curve
OR: Odds ratio
CI: Confidence interval
