# Supplementary material for: Impact of breast tumor size discrepancy between contrast-enhanced and conventional ultrasonography on axillary node metastasis: a retrospective cohort study
Source: BMC Cancer. 2025 Nov 5;25:1718. doi: 10.1186/s12885-025-15167-9 (PMC12587728; doi:10.1186/s12885-025-15167-9)
Supplement: Supplementary file 1 — Supplementary Material 1. [file 12885_2025_15167_MOESM1_ESM.docx]

Supplementary table CEUS findings of primary breast tumor

| **CEUS findings of primary breast tumor** | **Positive finding** |
| --- | --- |
| A1: Enhanced time compared with surrounding breast tissue; earlier, synchronous, or later | Earlier |
| A2: Enhanced intensity compared with surrounding tissue; enhanced or not | Enhanced |
| A3: Enhanced direction; centripetal, centrifugal, or diffuse enhancement | Centripetal |
| A4: Internal homogeneity of the lesion; heterogeneous or homogeneous | Heterogeneous |
| A5: Margin of the lesion after enhancement; clear or not clear | Not clear |
| A6: Shape of the lesion; regular or irregular | Irregular |
| A7: Ring-like enhancement; present or absent | Present |
| A8: Scope of the lesion (both long and short diameter of the lesion in the contrast-enhanced ultrasonography image compared to the one in conventional ultrasonography image); extended or not extended | Extended |
| A9: Perfusion defect; present or absent | Present |
